# Mitophagy in Alzheimer’s Disease and Other Age-Related Neurodegenerative Diseases

**DOI:** 10.3390/cells9010150

**Published:** 2020-01-08

**Authors:** Qian Cai, Yu Young Jeong

**Affiliations:** Department of Cell Biology and Neuroscience, Rutgers, The State University of New Jersey, Piscataway, NJ 08854, USA

**Keywords:** mitophagy, mitophagosome, lysosome, mitochondrial dynamics, mitochondrial quality control, Alzheimer’s disease, Parkinson’s disease, Huntington’s disease, amyotrophic lateral sclerosis, aging

## Abstract

Mitochondrial dysfunction is a central aspect of aging and neurodegenerative diseases, including Alzheimer’s disease, Parkinson’s disease, amyotrophic lateral sclerosis, and Huntington’s disease. Mitochondria are the main cellular energy powerhouses, supplying most of ATP by oxidative phosphorylation, which is required to fuel essential neuronal functions. Efficient removal of aged and dysfunctional mitochondria through mitophagy, a cargo-selective autophagy, is crucial for mitochondrial maintenance and neuronal health. Mechanistic studies into mitophagy have highlighted an integrated and elaborate cellular network that can regulate mitochondrial turnover. In this review, we provide an updated overview of the recent discoveries and advancements on the mitophagy pathways and discuss the molecular mechanisms underlying mitophagy defects in Alzheimer’s disease and other age-related neurodegenerative diseases, as well as the therapeutic potential of mitophagy-enhancing strategies to combat these disorders.

## 1. Introduction

Mitochondria are termed the “powerhouses” of the cell, and generate the majority of the cell’s supply of adenosine triphosphate (ATP) through the oxidative phosphorylation system (OXPHOS) in which electrons produced by the citric acid cycle are transferred down the mitochondrial respiratory complexes. Neurons have particularly high and continuous energy demands so that mitochondrial function is essential for maintaining neuronal integrity and responsiveness [1,2,3,4,5,6,7]. Mitochondrial energy production fuels various critical neuronal functions, especially the ATP-dependent neurotransmission [1,3,8]. Along with regulating energy levels, mitochondria have a high capacity to sequester excessive Ca^2+^ and release Ca^2+^ so as to prolong residual levels at synaptic terminals [9,10]. Through this mechanism, mitochondria play essential roles in maintaining and regulating neurotransmission [11,12], as well as certain types of short-term synaptic plasticity [13,14]. In addition, mounting evidence has demonstrated the critical role of mitochondria in the maintenance of cellular homeostasis [15]. Glucose was shown to be an efficient energy source in neurons and glia that can consume energy produced in parallel by glycolysis and OXPHOS. However, upon neural network activation, the energy demand is robustly enhanced. Given ATP as the main energy source in neurons, mitochondrial energy metabolism thus may play a major role in supplying ATP to fuel these neuronal activities. Importantly, distinct mitochondrial energetic status might also have a significant impact on the cellular signaling pathways. Aged and dysfunctional mitochondria are defective in ATP production and Ca^2+^ buffering, leading to energy deficit and interruptions of neuronal function and health. Furthermore, damaged mitochondria trigger concomitant leakage of electrons and thus promote the production of harmful reactive oxygen species (ROS) that can damage nucleic acids, proteins, and membrane lipids [1,16,17,18]. Moreover, mitochondrial oxidative stress leads to the release of cytochrome *c*, a mitochondrial intermembrane space protein, into the cytosol, inducing DNA damage, caspase activation, and apoptosis [6].

A large body of work suggests that mitochondrial dysfunction underlies cognitive decline in neuronal aging and is one of the most notable hallmarks of age-associated neurodegenerative diseases. Mitochondrial damage causes energy deficit, oxidative stress, and impaired cellular signaling, which has been linked to the pathogenesis of neurodegeneration diseases [2,19,20,21]. Given that a mitochondrion’s half-life is estimated to be about 30 days [22,23], cells have developed the interconnected and elaborate pathways through the balance of mitochondrial biogenesis and efficient removal of damaged mitochondria to ensure the maintenance of mitochondrial integrity and bioenergetic functions. Mitophagy, a selective form of autophagy, constitutes a key pathway of mitochondrial quality control mechanisms involving sequestration of defective mitochondria into autophagosomes for subsequent lysosomal degradation [1,2,24,25]. Disruption of mitophagy has been indicated in aging and various diseases, including neurodegenerative disorders such as Alzheimer’s disease (AD), Parkinson’s disease (PD), amyotrophic lateral sclerosis (ALS), and Huntington’s disease (HD) [7,26]. This review aims to provide a thorough and timely overview of the mitophagy pathways, summarize the underlying mechanisms of mitophagy defects in AD and other age-related neurodegenerative diseases, and highlight the possible therapeutic strategies targeting mitophagy towards confronting mitochondrial dysfunction and neurodegeneration.

## 2. Overview of the Mitophagy Pathways

**Figure 1:**
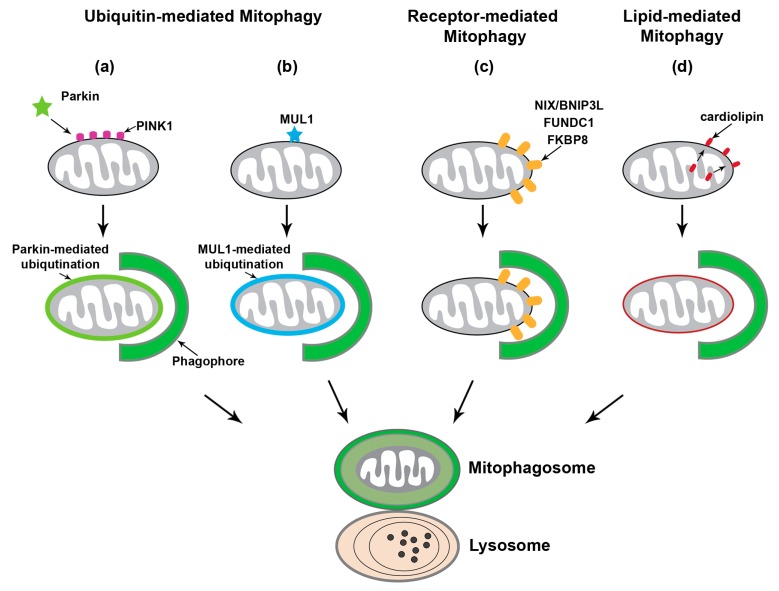
The mitophagy pathways. Upon mitochondrial damage, mitophagy can be induced through three major mechanisms: ubiquitin-mediated mitophagy including PTEN-induced putative kinase protein 1 (PINK1)-Parkin-dependent mitophagy, outer mitochondrial membrane (OMM) receptor-mediated mitophagy, and lipid-mediated mitophagy. (**a**) PINK1-Parkin-mediated mitophagy initiates with PINK1 stabilization on the OMM of damaged mitochondria to recruit Parkin, an E3 ubiquitin ligase. Phospho-ubiquitination of substrates on the OMM by PINK1/Parkin recruits the autophagy machinery and thus promotes the engulfment of damaged mitochondria by growing phagophore or isolation membranes. (**b**) Other E3 ubiquitin ligases have been reported to regulate mitophagy independent of PINK1 and Parkin. MUL1 has similar substrates to Parkin and can directly bind to GABAA receptor-associated protein (GABARAP), suggesting that MUL1 can function independently to facilitate autophagic engulfment. (**c**) Several autophagy receptors are anchored within the OMM, including NIP3-like protein X (NIX; also known as BNIP3L), FUN14 domain containing 1 (FUNDC1), and FK506 Binding Protein 8 (FKBP8). Binding of NIX/BNIP3L or FUNDC1 to LC3 or GABARAP on the phagophore mediates targeting dysfunctional mitochondria for autophagy. (**d**) Externalization of cardiolipin, normally found in the inner mitochondrial membrane (IMM) phospholipid, to the OMM is a unique mechanism for lipid-mediated mitophagy. Cardiolipin initiates mitophagy through its direct interaction with LC3.

Mitophagy (mitochondrial autophagy) is the only known cellular pathway through which entire mitochondria are completely eliminated within lysosomes. Under physiological conditions, mitophagy plays an essential role in the basal mitochondrial turnover and maintenance. More importantly, mitophagy can also be robustly induced in response to a variety of pathological stimuli [7,25,26,27]. There are a number of mitophagy pathways that have been identified (Figure 1).

### 2.1. PINK1-Parkin-Mediated Mitophagy

PTEN-induced putative kinase protein 1 (PINK1)-Parkin-mediated mitophagy is the most heavily studied and the best-understood mitophagy pathway [28,29,30]. In brief, loss of mitochondrial membrane potential (Δψ_m_) accumulates PINK1 on the outer membrane of mitochondria (OMM) to recruit and activate Parkin, an E3 ubiquitin ligase, through phosphorylation of ubiquitin [31,32,33,34,35,36,37,38]. Parkin then ubiquitinates a number of OMM proteins and subsequently activates the ubiquitin-proteasome system (UPS) to degrade these ubiquitinated OMM proteins [39,40,41,42,43]. This leads to recruitment of the autophagy machinery to promote the engulfment of damaged mitochondria by phagophore or isolation membranes and thus formation of mitophagosomes destined for removal via the lysosomal system. The roles of PINK1 and Parkin in mitochondrial quality control and mitophagy have been supported by multiple studies in *Drosophila* [28,44,45,46,47]. The PINK1-Parkin pathway was shown to facilitate mitophagy as well as selective mitochondrial respiratory chain turnover [28,44,45,46,47]. Furthermore, genetic and clinical data have provided clear evidence to support the notion that the PINK1-Parkin pathway is involved in the pathogenesis of PD [48,49]. However, recent in vivo studies indicate that PINK1 and Parkin are not critical for basal mitophagy in a range of tissues including the brain [50,51]. More recent studies have been focused on understanding the PINK1-Parkin-independent mitophagy pathways.

### 2.2. Ubiquitin-Mediated Mitophagy Independent of Parkin

Other E3 ubiquitin ligases that can also mediate removal dysfunctional mitochondria have been identified [52], which is with relation to PINK1-Parkin-independent mitophagy mechanisms. Mitochondrial ubiquitin ligase 1 (MUL1, also known as MAPL, GIDE, and MULAN) was reported to play a role in the regulation of mitophagy through multiple mechanisms. MUL1 interacts with mitochondrial fission GTPase protein dynamin-related protein 1 (Drp1) and mitochondrial fusion protein Mitofusin, both of which are the substrates of Parkin [53,54]. MUL1 has no effect on PINK1-Parkin-mediated mitophagy, but can suppress PINK1 or Parkin mutant phenotypes in both *Drosophila* and mouse neurons. This suppression is attributed to the ubiquitin-dependent degradation of Mitofusin. Interestingly, double mutants of MUL1 with either PINK1 or Parkin show much more severe phenotypes. Moreover, MUL1 contains an LC3-interacting region (LIR) motif in the RING domain through which MUL1 interacts with GABAA receptor-associated protein (GABARAP), a member of the Atg8 family that plays a key role in autophagy and mitophagy [55]. Thus, these observations collectively suggest that MUL1 functions in a pathway parallel to the PINK1-Parkin pathway. In addition to MUL1, a recent study reported PINK1-synphilin-1-SIAH-1 as another newly discovered Parkin-independent pathway that can promote PINK1-dependent mitophagy in the absence of Parkin [56].

### 2.3. Receptor-Mediated Mitophagy

The BCL-2 homology 3 (BH3)-containing protein NIP3-like X (NIX, also known as BNIP3L), an OMM protein, was reported to play an important role in mitochondrial turnover in erythrocytes [57]. NIX/BNIP3L contains an LIR motif at the amino-terminal that binds to LC3 on phagophore or isolation membranes, and is transcriptionally upregulated during erythrocyte differentiation [58]. Such a mechanism enables NIX/BNIP3L to serve as a selective mitophagy receptor and promote recruitment of the autophagy machinery to the surface of damaged mitochondria in erythroid cells. NIX/BNIP3L was also reported to be involved in hypoxia-induced mitophagy, during which forkhead box O3 (FOXO3) and hypoxia-inducible factor (HIF) transcriptionally regulate NIX/BNIP3L along with BNIP3 [59]. Noteworthy, overexpression of NIX/BNIP3L can restore mitophagy in skin fibroblasts from PD patients carrying mutations in *PARK6* or *PARK2* [60], suggesting an independent role of NIX/BNIP3L in PINK1-Parkin-mediated mitophagy. NIX/BNIP3L and BNIP3 were reported to be upregulated upon neuronal stress [61,62]. However, the extent to which NIX/BNIP3L and BNIP3 might participate in neuronal mitophagy remains unclear. FUN14 domain containing 1 (FUNDC1) also functions as a mitophagy receptor and regulates the autophagic clearance of mitochondria under hypoxic stress. Studies have demonstrated that the mitochondrial phosphatase phosphoglycerate mutase family member 5 (PGAM5) dephosphorylates FUNDC1 to activate mitophagy during hypoxia [63,64,65]. Additionally, FK506 Binding Protein 8 (FKBP8) was recently reported to have LIR domains and can mediate Parkin-independent mitophagy by recruiting LC3A [66]. Collectively, these observations suggest that specific mitophagy receptors on the OMM play an essential role in recruiting the autophagy machinery to damaged mitochondria for lysosomal clearance.

### 2.4. Lipid-Mediated Mitophagy

Recent studies have demonstrated that lipids can also act as an elimination signal to mediate recruitment of injured mitochondria to the autophagy pathway. Apart from ubiquitin- or receptor-mediated mitophagy, this pathway involves the direct interaction of LC3 with the phospholipid cardiolipin, and was originally observed in neuroblastoma cells and primary cortical neurons incubated with rotenone, staurosporine, or 6-hydroxydopamine [67]. Cardiolipin is primarily found in the inner membrane of mitochondria (IMM) and is externalized to the OMM upon mitochondrial damage. Three enzymatic translocations are needed for the externalization of cardiolipin, which are mediated by the phospholipid scramblase-3 of mitochondria and the inner and outer membrane spanning hexameric complex of mitochondrial nucleoside diphosphate kinase D (NDPK-D/NM23-H4) in SH-SY5Y cells or Tafazzin (TAZ) in mouse embryonic fibroblasts (MEFs), respectively [67,68,69]. Furthermore, cardiolipin interacts with LC3, and this interaction is facilitated by the negatively charged basic residues in LC3 and charged head group of cardiolipin. Thus, cardiolipin-mediated mitophagy is independent of PINK1 and Parkin. Importantly, cardiolipin downregulation or mutagenesis of LC3 at the sites predicted to interact with cardiolipin was shown to impair mitophagosome formation [67]. In addition, genome-wide screens indicate that F-box and WD40 domain protein 7 (FBXW7), sterol regulatory element binding transcription factor 1 (SREBF1), and other components of the lipogenesis pathway may play a role in the regulation of Parkin-mediated mitophagy [70]. Additionally, upon Drp1-mediated mitochondrial fission, ceramide was shown to promote autophagic recruitment of mitochondria through direct interaction of ceramide with LC3B-II [71].

### 2.5. Neuronal Mitophagy

**Figure 2:**
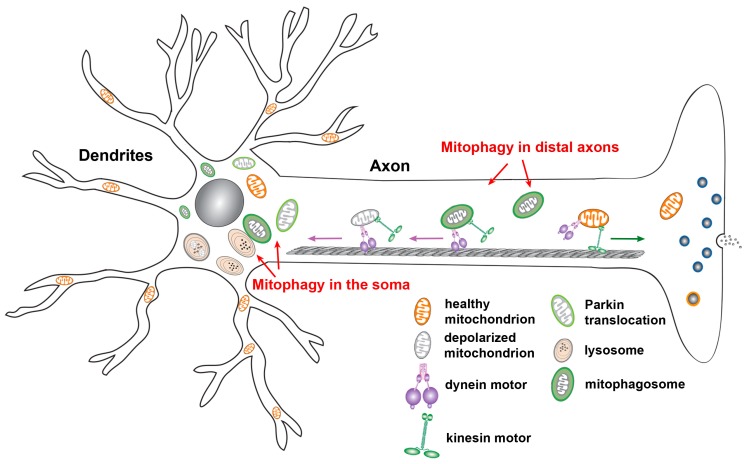
Neuronal mitophagy. Upon mitochondrial membrane potential (Δψ_m_) dissipation, Parkin is recruited to depolarized mitochondria, triggering mitochondrial engulfment by autophagosomes. Parkin-targeted mitochondria accumulate in the somatodendritic regions of neurons. Such compartmental restriction is attributed to altered mitochondrial motility along axons, as evidenced by decreased anterograde transport and relatively increased retrograde transport of mitochondria. This spatial process allows neurons to efficiently remove damaged mitochondria from axonal terminals and facilitate mitophagic clearance in the soma, where mature lysosomes are mainly located. Studies have also shown that autophagosomes containing engulfed mitochondria move in an exclusively retrograde direction from distal axons toward the soma for maturation and for more efficient cargo degradation within acidic lysosomes in the soma. Figure is modified from [2].

Neurons are highly polarized cells with unique properties in structure and function. Mitochondrial quality control mechanisms that efficiently sense and eliminate mitochondria damaged over usage, aging, or disease could be critical for neuronal health. Mitophagy is currently believed to constitute the major cellular pathway for mitochondrial quality control in neurons. While basal mitophagy is known to be required for the maintenance of neuronal homeostasis, mounting evidence has shown that mitophagy can be upregulated in response to various pathological stimuli (Figure 2). Cardiolipin-mediated mitophagy can be induced in primary cortical neurons treated with the mitochondrial complex I inhibitor rotenone [67]. As for Parkin-mediated mitophagy, Δψ_m_ dissipation triggers Parkin translocation onto depolarized mitochondria in neurons after treatment with CCCP, an Δψ_m_ uncoupler [72,73]. Interestingly, Parkin-targeted mitochondria primarily accumulate in the somatodendritic region of neurons where they undergo autophagic sequestration for lysosomal degradation. Moreover, mitophagy activation reduces anterograde transport, but increases retrograde transport of axonal mitochondria, suggesting that damaged mitochondria are trafficked back to the soma for mitophagic clearance. Parkin-dependent mitophagy was also discovered under AD-linked pathophysiological conditions in the absence of any Δψ_m_ dissipating reagent [74]. The spatial aspects of Parkin-dependent mitophagy were also observed in vivo. In particular, the PINK1 and Parkin mutant *Drosophila* exhibit abnormal tubular and reticular mitochondria restricted to the cell body, as well as normal morphology with reduced mitochondrial flux within axons [46,47]. In addition to *Drosophila*, the evidence from examination of Purkinje neurons in the mito-QC reporter mice suggests that the majority of mitochondrial turnover occurs in the Purkinje somata. This supports the view that damaged mitochondria or mitophagosomes are returned to the cell body for lysosomal clearance [75]. Collectively, these in vitro and in vivo observations consistently suggest that the soma is in the focus of neuronal mitophagy, a selective process with a function to restrict damaged mitochondria to the soma and thus limit the impact of impaired mitochondrial function on distal axons.

### 2.6. Mitophagy In Vivo

The mitophagy pathways have been extensively studied *in vitro*. To address the basal mitophagy in vivo, a number of transgenic mice expressing sensors to monitor the delivery of mitochondria to acidic organelles (lysosomes) have been developed [75,76]. These studies have demonstrated active mitochondrial delivery to acidic organelles in multiple tissues but with variable rates. A recent work further shows that the basal mitophagy is independent of the PINK1 pathway [51]. Consistently, studies from *Drosophila* expressing fluorescent mitophagy reporters, either mito-Keima or mito-QC, also reveal robust basal mitophagy in different tissues [50]. However, null mutations of either PINK1 or Parkin do not lead to altered rates of mitochondrial delivery into lysosomes, suggesting nonessential roles of PINK1 and Parkin in the basal mitophagy in vivo. These data are also consistent with the observations from mice with the deletion of *PARK6* or *PARK2*. These mice lack strong phenotypes, such as dopaminergic neuron loss [77,78,79,80,81]. Importantly, the evidence of mitophagy activation is clear in the brain tissues of human patients with neurodegenerative diseases [74,82,83]. Given multiple distinct mechanisms that have been identified to target damaged mitochondria for autophagy, other PINK1-Parkin-independent pathways or other as yet undefined mechanisms likely play more important role in the basal neuronal mitophagy. Therefore, the involvement of these mitophagy pathways in the basal mitochondrial turnover and in response to specific disease-related stressors needs to be carefully determined in vivo.

## 3. Mitochondrial Dysfunction in Neurodegenerative Diseases

**Figure 3:**
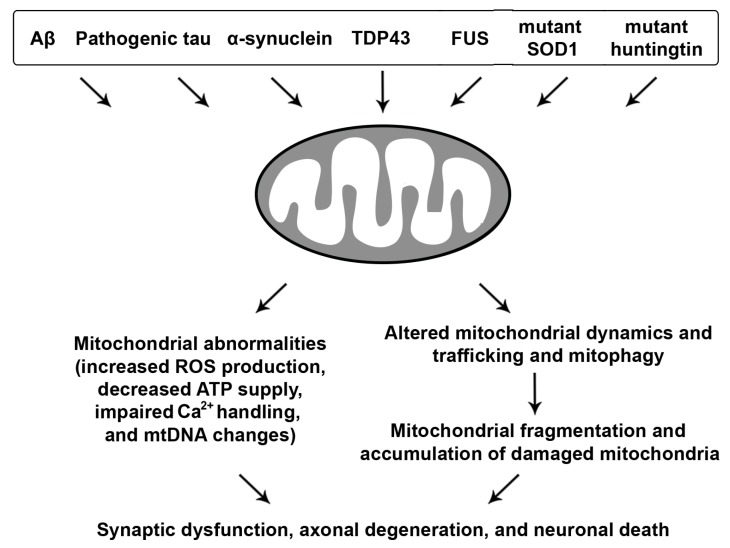
Neurodegenerative disease-associated mitochondrial toxicity. Misfolded proteins, oligomers, protein aggregates, or fibrils linked to major neurodegenerative diseases induce mitochondrial abnormalities, leading to increased reactive oxygen species (ROS) levels, loss of mitochondrial membrane potential (Δψ_m_), decreased oxidative phosphorylation (OXPHOS) and ATP production, impaired Ca^2+^ buffering, and enhanced mitochondrial DNA (mtDNA) changes. Moreover, impairments in mitochondrial dynamics and trafficking as well as mitophagy result in excessive mitochondrial fission and fragmentation and aberrant accumulation of dysfunctional mitochondria, all of which collectively contribute to synaptic dysfunction, axonal degeneration, and neuronal death.

Mitochondrial defects are a significant concern in the aging nervous system and have been consistently linked to age-related neurodegenerative diseases, suggesting that the underlying mechanisms might be somewhat shared (Figure 3).

### 3.1. Aβ and Tau-Linked Mitochondrial Abnormalities

AD is the most common form of neurodegenerative diseases in aging populations. Progression of the disease involves cognitive decline, memory loss, and neuronal death in the cerebral cortex and subcortical regions. AD patient brains are characterized by extracellular amyloid plaque deposits, composed of agglomerated amyloid β (Aβ) peptides, as well as intracellular accumulation of neurofibrillary tangles (NFTs), consisting of hyperphosphorylated tau (phospho-tau) protein. Mitochondrial disturbances have been suggested as a hallmark of AD as the patients exhibit early metabolic alterations prior to any histopathological or clinical manifestations [84]. Mitochondrial dysfunction, oxidative stress, and mitochondrial DNA (mtDNA) changes are prominent pathological features reported in AD postmortem brains [85,86,87,88,89,90,91,92,93,94,95]. Importantly, a growing body of evidence has indicated a major role of mitochondrial defects in the pathogenesis of AD [2,96,97,98].

The degree of cognitive dysfunction in AD was linked to the extent of Aβ accumulation within mitochondria and mitochondrial abnormalities [99]. Aβ has been proposed to be a key player in mediating mitochondrial damage. Aβ was found to impair multiple aspects of mitochondrial function [100,101,102], including function of the electron transport chain (ETC) [103], ROS production [104,105,106], mitochondrial dynamics [91,103,107,108], and mitochondrial transport [109,110,111]. The possible routes for Aβ to enter into mitochondria were thought to be through the translocase of the outer membrane (TOM) complex or mitochondrial-associated endoplasmic reticulum (ER) membrane (MAM) [112,113,114,115]. In addition to intracellular Aβ, mitochondria can also take up internalized extracellular Aβ [114,116]. Aβ1–42 treatment was shown to lead to the opening of mitochondrial permeability transition pore (mPTP) in cultured cortical neural progenitor cells. While transient mPTP opening decreases cell proliferation, prolonged mPTP opening irreversibly causes cell death [117]. Consistent with this observation, an interesting work in a live AD mouse model provided direct evidence that fragmented and defective mitochondria are limited to the vicinity of extracellular amyloid plaques that likely serve as a focal source to promote abnormal accumulation of Aβ within mitochondria and thus exacerbate Aβ-linked damage [118].

The mechanisms underlying Aβ-mediated mitochondrial toxicity have been carefully investigated by several studies. The interactions of Aβ with Aβ-binding alcohol dehydrogenase (ABAD), a mitochondrial matrix protein, and cyclophilin D (CypD), a component of the mitochondrial transition pore, were reported to mediate Aβ-induced cytotoxic effects [101,102]. In particular, ABAD was shown to be upregulated in AD neurons. Overexpression of ABAD can exacerbate Aβ-induced cellular oxidative stress and cell death. Aβ also forms a complex with CypD in the cortical regions of postmortem human AD patient brains and an AD mouse model [102]. Deletion of CypD in AD mice rescues the mitochondrial phenotypes including impaired Ca^2+^ uptake, mitochondrial swelling due to increased Ca^2+^, depolarized Δψ_m_, elevated oxidative stress, decrease in ADP-induced respiration control rate, and reduced complex IV activity and ATP levels. Moreover, CypD deficiency can improve synaptic function as well as learning and memory in an AD mouse model [102]. These observations collectively suggest that Aβ-CypD interaction mediates AD-associated mitochondrial defects. Taken together, these pieces of evidence indicate that the aberrant accumulation of Aβ within mitochondria likely plays a causative role in impaired mitochondrial function in AD.

Pathogenic forms of tau can also induce mitochondrial damage. A number of studies have demonstrated that phospho-tau specifically impairs complex I of the mitochondrial respiratory chain, resulting in increased ROS production, loss of Δψ_m_, lipid peroxidation, and reduced activities of detoxifying enzymes such as superoxide dismutase (SOD) [119,120]. Overexpression of the mutant human tau protein htauP301L was reported to reduce ATP levels and increase susceptibility to oxidative stress in cultured neuroblastoma cells [121]. Disrupted activity and altered composition of mitochondrial enzymes can also be detected in the P301S mouse model of tauopathy [122]. In the pR5 mice overexpressing the htauP301L, mitochondrial dysfunction was evidenced by impaired mitochondrial respiration and ATP synthesis, decreased complex I activity, and increased ROS levels [123,124]. Phospho-tau was also reported to directly interact with VDAC in AD brains. This interaction was proposed to impair mitochondrial function likely through blocking mitochondrial pores [125]. Furthermore, mitochondrial stress was shown, in turn, to enhance hyperphosphorylation of tau in a mouse model lacking SOD2 [126]. Inhibition of mitochondrial complex I activity reduces ATP levels, resulting in a redistribution of tau from the axon to the soma and subsequent cell death [127]. Thus, these observations suggest that the toxic effects of tau on mitochondria could be reciprocal and that mitochondrial deficiency might play a critical role in the development of tau pathology.

Both Aβ and pathogenic tau have deleterious effects on mitochondrial dynamics through which impact mitochondrial function. Studies on postmortem brain tissues from human patients with AD and mouse models have demonstrated increased levels of Drp1 and Fis1 and reduced levels of mitofusin 1 (Mfn1), Mfn2, and OPA1. Moreover, Aβ overproduction, phospho-tau accumulation, as well as abnormal interactions of Drp1 with Aβ or phospho-tau cause excessive mitochondrial fission and fragmentation, which tend to increase as AD progresses [91,97,108,125,128]. Cells overexpressing mutant tau associated with frontotemporal dementia (FTD) with Parkinsonism linked to chromosome 17 (FTDP-17) display decreased rates of mitochondrial fusion and fission and enhanced vulnerability to oxidative stress [121]. Strikingly, reduction of Drp1 expression can protect against mutated tau-induced mitochondrial dysfunction [129]. Collectively, these data suggest that the pathogenic forms of tau and Aβ could impair mitochondrial function either through direct interaction with VDAC, ABAD, or CypD, or indirectly through their toxic effects on mitochondrial dynamics.

### 3.2. Mitochondrial Defects with Synucleinopathies

PD is the second most common form of neurodegenerative disease, which is characterized by the aberrant accumulation of α-synuclein (α-syn) in the form of Lewy bodies, especially in the substantia nigra. α-syn is abundant throughout the central nervous system and Lewy bodies are a defining feature of many clinical phenotypes known as synucleinopathies [130,131]. Importantly, impaired mitochondrial function is also a pathological feature of both sporadic and familial PD [20,28,44,49,132,133,134]. The relationship between α-syn and mitochondria has been explored in many studies. Some evidence showed that mitochondria could be the main targets of α-syn. In particular, the oligomerization and aggregation of α-syn can cause deficits in the complex I activities, leading to reduced ATP levels, depolarized Δψ_m_, and the release of cytochrome *c* into the cytosol to trigger apoptosis [135,136]. A number of studies have shown that α-syn is directly localized in mitochondria, and can be detected in isolated mitochondria from PD patient brains. Mitochondrial localization of α-syn has a negative impact on mitochondrial function, morphology, and dynamics [137,138,139,140,141,142,143]. α-syn has a cryptic mitochondrial targeting sequence located at its amino terminal region through which α-syn is constitutively imported into mitochondria and associates with the IMM. Such a mechanism leads to reduced complex I activities and elevated ROS levels in human dopaminergic neurons [140]. Moreover, oligomeric and dopamine-modified α-syn disrupts the association of the OMM translocase TOM20 and its coreceptor, TOM22, resulting in protein import impairment [144]. Thus, diminished import of mitochondrial proteins impairs mitochondrial function in nigrostriatal neurons, as reflected by deficient respiration, loss of Δψ_m_, and enhanced production of ROS.

α-syn can also affect mitochondrial dynamics and mitophagy. Basically, α-syn is known to bind to the lipid membranes, especially the lipids of the ER membrane or the MAM through which ER interacts with mitochondria. Mutated α-syn decreases the ER-mitochondria contact or interaction, leading to MAM dysfunction and thus mitochondrial fragmentation [141]. Other studies into PD have demonstrated that α-syn causes mitochondrial fragmentation through either direct binding or as a result of increased Drp1 [142,145]. Cleavage of Opa1 was found in dopaminergic neurons with overexpression of α-syn, resulting in decreased mitochondrial fusion [145]. Consistently, suppression of Drp1-mediated mitochondrial fission was reported to protect cells from α-syn-induced cytotoxicity [146]. In addition, studies have demonstrated the direct binding of α-syn to cardiolipin [147,148]. Furthermore, PD-related SNCA-mutant neurons exhibit increased externalization of cardiolipin to the OMM. Externalized cardiolipin was shown to bind to and promote refolding α-syn fibrils. Importantly, the exposed cardiolipin initiates LC3 recruitment to mitochondria and thus enhances mitophagic turnover, leading to reduced mitochondrial volume and exacerbation of mutant α-syn-induced mitochondrial dysfunction [148]. On the other hand, mitophagy defects were also proposed to play a significant role in PD pathogenesis, especially augmenting α-syn accumulation and its mediated neurotoxicity [149,150,151].

### 3.3. ALS and FTD-Associated Mitochondrial Toxicity

ALS is a devastating disease characterized by motor neuron degeneration. A hallmark of ALS, as in the pathologies of other neurodegenerative diseases, is the abnormal accumulation of misfolded proteins and protein aggregates within the affected motor neurons. FTD affects the basal ganglia and cortical neurons, leading to cognitive deficits, language deficiency along with altered social behavior and conduct. Even though the affected neuron types are quite different, ALS and FTD show the similarities in genetic background and pathological processes and also share the common pathways of neurodegeneration [152,153]. Defects in oxidative phosphorylation, Ca^2+^ buffering, and increased ROS production have been linked to ALS pathogenesis [154]. Multiple studies in cell culture and in transgenic animal models of ALS reveal alterations in oxidative metabolism linked to changes in ETC activity and reduced ATP synthesis [155,156,157,158]. More importantly, mitochondria purified from ALS patients display impaired Ca^2+^ homeostasis and increased ROS levels. Such defects are coupled with oxidative damage including altered tyrosine nitration and protein carbonylation [159,160]. Indeed, glutamate-receptor mediated excitotoxicity was linked to overloaded mitochondrial Ca^2+^ and increased ROS levels in spinal motor neurons cultured from an ALS animal model [161].

Aggregation of the transactive response DNA-binding protein 43 kDa (TDP-43) and fused in sarcoma (FUS) is the pathological hallmarks of both ALS and FTD. Both TDP-43 and FUS are ribonuclear proteins and contain the glycine molecule-enriched prion-like domains that can increase the propensity of TDP-43 and FUS for aggregation as well as cell-to-cell transmission. Aged animals expressing mutant FUS exhibit abnormal accumulation of ubiquitin-positive aggregates, which correlates with neuron loss. These aggregates also stain positive for mitochondrial protein cytochrome *c*, suggesting that damaged mitochondria recruit the autophagy machinery for removal through mitophagy [162]. One study from a single postmortem analysis of an FUS mutation carrier uncovered similar defects. Additionally, C- and N-terminal fragments of TDP-43 were identified within mitochondria. Furthermore, animal models of TDP-43 pathology exhibit membranous organelle redistribution and clustering within cytoplasmic inclusions accompanied by morphological and ultrastructural alterations, as well as abnormal mitochondrial dynamics, trafficking, and quality control [163,164,165]. Thus, these data consistently indicate the phenotypes of mislocalized, fragmented, and defective mitochondria associated with ALS and FTD.

### 3.4. Mutant Htt-Induced Mitochondrial Damage

HD is a neurodegenerative genetic disease that affects muscle coordination and leads to cognitive decline and psychiatric symptoms [166]. This autosomal dominant inherited neurodegenerative disease is the most common genetic cause of abnormal involuntary movements called chorea, and is characterized by mutations in the huntingtin gene (*HTT*) that result in abnormal expansion of the cytosine–adenine–guanine (CAG) trinucleotide repeats in the *HTT* gene, encoding a polyglutamine (polyQ) tract at the N-terminal region of the huntingtin (Htt) protein. The N-terminus of Htt can be cleaved through protease activity, leading to formation of short and toxic polyQ peptides. The N-terminal fragments of Htt containing the polyQ tracts are more prone to aggregation and accumulate within the inclusions in the nucleus especially in the medium spiny neurons of the striatum [167,168,169,170,171]. Htt was reported to directly bind to Tim 23 on mitochondria, thus preventing the protein import into mitochondria. This defect could be reversed through overexpressing Tim 23 [172,173]. In addition, the aggregate accumulation can disrupt the ETC function [174]. Moreover, studies in HD patient brains found decreased activities of complex II, complex III, and complex IV. Reduced complex II activity was observed particularly in the striatum of HD patients. Such defects along with reduced ATP production were also demonstrated in other studies, which collectively point towards impaired OXPHOS and disrupted mitochondrial energy metabolism [175]. Importantly, overexpression of complex II reduces the mutant Htt-mediated toxic effects in striatal neurons. Moreover, alterations in mitochondrial dynamics were also reported in the striatum of HD patients, as well as in animal and cell models [176,177]. Such a defect is caused by abnormal interaction of mutant Htt with Drp1, leading to Drp1-enhanced mitochondrial fission and thus mitochondrial fragmentation as well as cellular dysregulation and death [178].

## 4. Mitophagy Defects in Neurodegenerative Diseases

**Figure 4:**
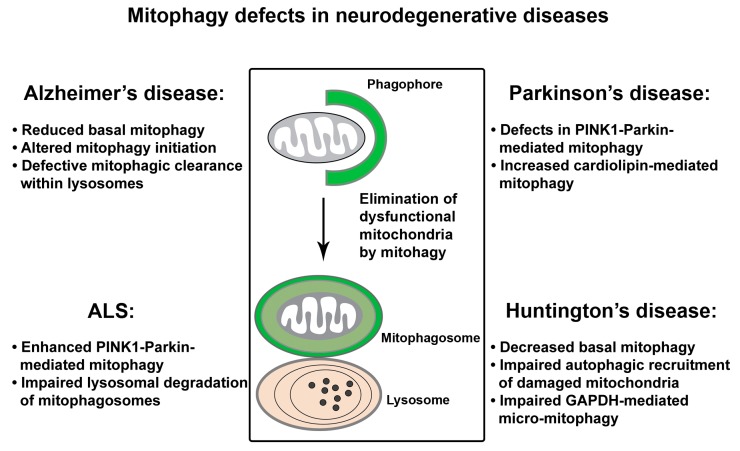
Mitophagy defects in neurodegenerative diseases. A growing body of evidence indicate that mitophagy function is impaired in major neurodegenerative diseases. In Alzheimer’s disease (AD), robust activation of Parkin-mediated mitophagy was observed in patient brains of familiar and sporadic AD and the cell and animal models. However, under tauopathy conditions, excessive or defective Parkin-dependent mitochondrial turnover was reported in different studies, respectively. In addition, impaired lysosomal proteolysis and reduced levels of the basal mitophagy collectively contribute to mitophagy dysfunction in AD. In Parkinson’s disease (PD), PINK1-Parkin-dependent mitophagy is necessary for the function and survival of PD-related dopaminergic neurons in response to stress/pathological stimuli. The role for increased cardiolipin-mediated mitophagy in mutant α-syn-induced mitochondrial dysfunction has also been proposed. As for amyotrophic lateral sclerosis (ALS), enhanced Parkin-mediated mitophagy was demonstrated in an ALS mouse model. Mitophagy defects and mitochondrial pathology are also attributed to lysosomal deficit in ALS affected motor neurons. In Huntington’s disease (HD), decreased basal levels of mitophagy, defects in autophagic recognition and recruitment of damaged mitochondria, and impaired GAPDH-mediated micro-mitophagy lead to pathological mitophagy and mitochondrial dysfunction.

Neurons have very high demand for ATP. Given that mitochondria are the major producer of ATP within cells, the nervous system is especially sensitive to mitochondrial damage. Inefficient elimination of injured mitochondria through mitophagy could be detrimental to neuronal health. Mitophagy deficit has been indicated in aging and the pathogenesis of age-associated neurodegenerative disorders. Studies into mitophagy suggest that defective mitophagy contributes to impaired mitochondrial function and neurodegeneration (Figure 4).

### 4.1. Mitophagy Defects in AD

Earlier studies revealed abnormal mitophagy in AD patient brains, as evidenced by autophagic accumulation of mitochondria in the soma of vulnerable AD neurons [87,179,180]. Among the multiple distinct mitophagy pathways, PINK1-Parkin-dependent mitophagy has been the focus of current studies in AD. We have shown that the Parkin pathway is robustly induced upon progressive Aβ accumulation and mitochondrial damage in human patient brains and animal models of AD [74]. Furthermore, cytosolic Parkin is depleted in AD brains over the disease’s progression, resulting in mitophagic pathology and augmented mitochondrial defects. Consistently, in the AD patient-derived skin fibroblasts and brain biopsies, another study reported diminished Parkin along with abnormal PINK1 accumulation [181]. Mitophagy can be restored in these cells by overexpression of Parkin, as reflected by decreased PINK1 and the recovery of Δψ_m_ coupled with reduced retention of defective mitochondria. Therefore, these findings indicate that impaired mitochondrial function and abnormal retention of dysfunctional mitochondria could be attributed to mitophagy defects in AD neurons. In addition, cardiolipin cluster-organized profile was shown to be lost in synaptic mitochondria purified from AD mouse models [182], occurring at the early disease stages and before nonsynaptic mitochondrial defects. This data suggests the possibility that cardiolipin-mediated mitophagy might be deficient in AD.

The degradation capacity of lysosomes is critical for mitophagic clearance, and defects in lysosomal proteolysis of autophagic cargoes can also impair the mitophagy function. Lysosomal deficit is a prominent feature in AD brains, linked to the pathogenesis of AD. Suppression of lysosomal proteolysis in wild-type (WT) mice was shown to mimic neuropathology of AD and exacerbate autophagic pathology and amyloidogenesis in AD mouse models [183,184]. Presenilin 1 mutations along with ApoE4, a key genetic risk factor of AD, are thought to disrupt lysosomal function [185]. Other factors, including Aβ peptides, phospho-tau, ROS, and oxidized lipids and lipoproteins, could also impair lysosomal proteolysis and result in a toxic accumulation, thus triggering apoptosis and neuronal death in AD. Our recent study proposes that AD-linked lysosomal deficit is also attributed to defects in protease targeting to lysosomes [186]. It is known that newly synthesized protease precursors need to be delivered from the trans-Golgi network (TGN) to the endo-lysosomal system for maturation, a process that relies on the presence of cation-independent mannose 6-phosphate receptor (CI-MPR) at the Golgi. The retromer complex mediates the retrieval of CI-MPR from late endosomes to the TGN and thus facilitates the trafficking of proteases to late endosomes and lysosomes [187]. Our study reveals that retromer dysfunction and defective CI-MPR recycling to the Golgi lead to defects in protease targeting to lysosomes. As a result, protease deficit within lysosomes impedes lysosomal proteolysis of defective mitochondria along with other autophagic cargoes in AD neurons [186]. Therefore, increased Parkin association with mitochondria, autophagic accumulation, as well as abnormal mitochondrial retention within lysosomes observed in AD neurons of patient brains and in cultured cells overexpressing mutant APP could also represent lysosomal deficiency [74,186]. Taken together, these observations indicate that defective mitophagy is likely involved in AD-linked neurodegeneration.

Pathogenic truncation of tau could impair mitophagy function. A recent work reported a stable association of an NH2-htau fragment with Parkin and Ubiquitin-C-terminal hydrolase L1 (UCHL-1) in cellular and animal AD models and human AD brains, leading to enhanced mitochondrial recruitment of Parkin and UCHL-1 and thus improper mitochondrial turnover [188]. Mitophagy suppression can restore synaptic mitochondrial density and partially, but significantly, protect against neuronal death induced by this NH2-htau. In contrast, another study proposed that human wild type tau (htau) is inserted into the mitochondrial membrane, thus inducing mitophagy impairment [189]. However, in a more recent study, both htau and htauP301L were shown to impair mitophagy in *Caenorhabditis elegans* (*C. elegans*) and neuroblastoma cells by reducing Parkin translocation onto mitochondria through a different mechanism. Instead of changes in the Δψ_m_ or the cytoskeleton, impaired Parkin recruitment to mitochondria is proposed to be caused by tau-mediated sequestration of Parkin in the cytosol [190]. Collectively, these data suggest that mitophagy is impaired under tauopathy conditions by distinct mechanisms.

In addition to defects in mitophagic clearance in response to Aβ and tau-induced mitochondrial damage, a recent study reveals a marked decrease in the basal level of mitophagy in postmortem hippocampal tissues from AD patients, cortical neurons derived from AD-induced pluripotent stem cell (iPSC), as well as AD mouse models [191]. This study further demonstrates defects in the activation of ULK1 and TBK1, the autophagy proteins that mediate autophagy/mitophagy initiation, thus leading to impaired mitophagy function. Furthermore, pharmacological reinstallation of mitophagy mitigates amyloid and tau pathologies, resulting in beneficial effects against memory loss in these AD mice. Therefore, these data support the view that defective mitophagy is likely an early event in AD brains and plays a causative role in the development of AD-linked neuropathology [191]. Further studies using neurons derived from iPSCs of sporadic AD or other similar models could be very critical to addressing whether mitophagy dysfunction serves as a key player in Aβ/tau proteinopathies.

### 4.2. Mitophagy Defects in PD

Dysfunctional mitophagy is closely linked to PD. Many PD-causing genes show the mitochondrial phenotypes [28,137,150,192,193,194,195,196,197]. In addition, PD patients have increased rates of mtDNA deletion in the substantia nigra, which further associates defective mitochondrial quality control with PD [198]. The important role for mitophagy in PD was first indicated from an ultrastructural study showing autophagic accumulation of mitochondria in neurons of the patients with PD and Lewy Body Dementia (LBD) [83]. Many other studies have demonstrated mitophagy abnormalities in a variety of experimental models representing genetic forms of toxic-environmental PD [67,199,200,201,202]. As previously discussed (see Section 2.1), cell-based and mechanistic studies directly link PINK1 and Parkin to mitophagy. However, while loss-of-function mutations in *PARK6* (encoding PINK1) and *PARK2* (encoding Parkin) genes are linked to familial PD [203], the role of the PINK1-Parkin-dependent pathway in vivo remains elusive. The PINK1 and Parkin pathway has been extensively studied in *Drosophila*. Mutant flies show dopaminergic degeneration, reduced lifespan, and locomotive defects [28,44,204,205,206]. Muscle cells of mutant flies exhibited swollen mitochondria with disrupted cristae, coupled with muscle degeneration [206,207,208,209]. *PARK6* KO rats showed dopaminergic loss and motor defects [210]. Importantly, both *Drosophila* and rat model systems show mitochondrial dysfunction. However, mice with the deletion of *PARK6* or *PARK2* do not exhibit robust substantial PD-relevant phenotypes [77,79,80,81]. This might be due to compensations by other mechanisms that are sufficient enough to maintain neuronal homeostasis under physiological conditions. Strikingly, when crossing *PARK2* KO mice with Mutator mice characterized by accelerated acquisition of mtDNA mutations, the resulting phenotypes have mitochondrial defects as well as dopaminergic neuronal loss [211]. Thus, this observation suggests that Parkin-mutant mice are susceptible to increased mtDNA damage. Given that both impaired mitochondrial function and mitophagy deficit are the upstream of neurodegeneration, the lack of robust phenotypes in mice suggest that the PINK1-Parkin pathway might be dispensable under physiological conditions, yet still necessary in response to stress/pathological stimuli for the functional maintenance and survival of PD-related dopaminergic neurons. Aside from PINK1-Parkin-dependent mitophagy, increased cardiolipin-mediated mitophagy was proposed to play a role in α-syn-induced mitochondrial dysfunction [148].

### 4.3. Mitophagy Defects in ALS

Impaired mitophagy was proposed to be involved in the denervation of neuromuscular junctions in an ALS mouse model [212]. Additionally, lysosomal dysfunction has been implicated in ALS. A recent work has provided a strong evidence showing that lysosomal deficits play a critical role in autophagy/mitophagy dysfunction and mitochondrial pathology in a mutant SOD1 transgenic mouse model of ALS [213]. Lysosomal deficits result in abnormal accumulation of autophagic vacuoles (AVs) engulfing damaged mitochondria within motor neuron axons of mutant SOD1 mice. More importantly, rescuing autophagy-lysosomal deficits was shown to enhance mitochondrial turnover, improve motor neuron survival, and ameliorate disease phenotype in mutant SOD1 mice. Given that autophagy/mitophagy is a lysosome-dependent pathway, defective mitophagy and mitochondrial pathology in ALS are attributed to defects in lysosomal proteolysis.

A more recent study uncovers that Parkin-dependent mitophagy is activated in the mutant SOD1 mouse model of ALS [214]. Mitophagy activation is known to induce Parkin-triggered and the UPS-mediated degradation of mitochondrial dynamics proteins Mfn2 and Mitochondrial Rho GTPase (Miro1) [42,215,216,217,218]. Consistently, increased mitophagy in the spinal cord of the mutant SOD1 mice is coupled with depletion of Parkin as well as mitochondrial dynamics proteins Mfn2 and Miro1 that are ubiquitinated by Parkin. Interestingly, genetic ablation of *PARK2* protects against muscle denervation and motor neuron loss and attenuates the depletion of mitochondrial dynamics proteins, which delays disease progression and prolongs life span in mutant SOD1 mice. Thus, the results from this study suggest that Parkin could be a disease modifier of ALS, and chronic activation of Parkin-dependent mitophagy augments mitochondrial dysfunction by depleting mitochondrial dynamics proteins. Consistently, several other studies also reported a significant reduction of Miro1 in spinal cord tissue of ALS patients and animal models [219]. Moreover, it was shown that Miro1 reduction induced by ALS-linked mutant SOD is dependent on Parkin [220]. Miro1 is known as a component of the adaptor-motor complex essential for KIF5 motors to drive anterograde transport of mitochondria along axons [8]. Miro1-knockout mice exhibit upper motor neuron degeneration [221]. Thus, ALS-linked mitochondrial trafficking defect is likely caused by Miro1 deficiency as a result of Parkin-dependent enhancement of Miro1 turnover [220].

Compromised mitophagy may also induce ALS. Many of the genes linked to ALS encode proteins that play a critical role in autophagy/mitophagy, including OPTN and p62, as well as their kinase TBK1 [222,223,224]. However, it is poorly understood how the mutations in these genes are involved in the ALS pathology. Given the phosphorylation of OPTN and p62 by TBK1 to activate autophagy/mitophagy, aberrant accumulation of misfolded proteins and protein aggregates along with impaired mitochondrial turnover may both contribute to ALS-linked mitochondrial dysfunction and motor neuron death. Illuminating the role of these proteins in vivo will be critical in dissecting the molecular and cellular mechanisms leading to axonal degeneration and motor neuron loss.

### 4.4. Mitophagy Defects in HD

Mitochondrial dysfunction and autophagy failure have been linked to the pathogenesis of HD. Mutant Htt is known to be associated with mitochondria and to mediate mitochondrial damage. Defective mitophagy may also be involved in mitochondrial defects in HD. Decreased levels of the basal mitophagy were shown in the dentate gyrus of HD mice crossed with the mito-Keima mouse line [76]. In addition to its role in catalyzing the sixth step of glycolysis, a recent study proposed that GAPDH functions in micro-mitophagy—the direct engulfment of injured mitochondria by lysosomes [225]. In HD cell models, abnormal interaction of long polyQ tracts with mitochondrial GAPDH impairs GAPDH-mediated mitophagy, leading to mitochondrial dysfunction and increased cell death. Additionally, mutant Htt can interact with and affect the autophagy machinery [226]. A primary defect in the ability of autophagosomes to recognize and recruit cytosolic cargoes was reported in HD cells, leading to inefficient autophagic engulfment of substrates including mitochondria. Such a defect contributes to HD-associated accumulation of abnormal mitochondria [227]. Moreover, Htt was proposed to act as a scaffold protein for autophagy through the physical interaction of Htt with p62 and ULK1 proteins. This interaction allows Htt to facilitate p62-mediated cargo recognition efficiency, in particular, associating Lys-63-linked ubiquitin-modified substrates with LC3-II—the integral component of phagophore or isolation membranes. Thus, this study supports the possibility that polyQ expansion might compromise the role of Htt in autophagy [228]. Given the evidence for HD-linked autophagy impairment and mitophagic pathology, investigations into mitophagy status as well as detailed mechanisms are important for better understanding of HD pathogenesis.

## 5. Mitophagy-Targeted Therapeutic Interventions

From the above stated, it is clear that mitochondrial damage is a hallmark of major neurodegenerative diseases. Pharmacological agents that induce mitophagy with a goal of enhancing clearance of damaged mitochondria could be a promising strategy for achieving a significant therapeutic benefit [7,98,229,230]. Several mitophagy inducers, including NAD^+^ precursors, urolithin A (UA), the antibiotic actinonin (AC), and spermidine [231,232], have been examined and shown significant benefits in enhancing mitophagy, increasing mitochondrial resistance to oxidative stress, prolonging health span, and for neuronal protection in disease animal models and/or human cells. The levels of NAD^+^ are reduced in AD animal models, and elevation of cellular NAD^+^ levels through supplementation with NAD^+^ precursors such as nicotinamide, nicotinamide mononucleotide (NMN), and nicotinamide riboside (NR) is found to attenuate Aβ and tau pathologies and protect against cognitive dysfunction [233]. Such beneficial effects are attributed to the enhancement of the NAD^+^-dependent SIRT1 and SIRT3, expression of the transcription factor CREB, and enhanced activities of PI3K-Akt and MAPK/ERK1/2 [191,233,234,235,236]. Additionally, NAD^+^ replenishment was also shown to restore mitochondrial function and thus ameliorate dopaminergic neuron loss in iPSC and *Drosophila* models of PD [237]. These observations collectively indicate that the interventions to sustain NAD^+^ levels might be beneficial for AD and PD patients.

UA is an ellagitannins-derived metabolite, and can effectively induce neuronal mitophagy in both *C. elegans* and mouse brains [191]. Both UA and AC-mediated mitophagy activation is dependent on PINK1, Parkin, and NIX, and was shown to attenuate AD pathologies, inflammation, and learning and memory deficits [191]. Polyamines, including spermidine, can increase autophagy activity through affecting autophagy-related gene expression as well as enhance mitophagy through the mechanisms of mammalian target of rapamycin (mTOR) inhibition and 5′ AMP-activated protein kinase (AMPK) activation [232,238]. Moreover, spermidine was shown to activate the Ataxia-Telangiesctasia mutated (ATM)-dependent PINK1/Parkin signaling. Treatment with spermidine can lead to memory improvement and prolonged life span observed in *C. elegans*, *Drosophila*, and mice [231,239].

Other pharmacological strategies to enhance mitophagy through inducing mild bioenergetic stress or inhibiting mTOR activity have also been proven to be beneficial in either delaying or treating AD. Mitochondrial uncoupling agents such as 2,4-dinitrophenol (DNP) were reported to stimulate autophagy and preserve neuronal function in AD animal models [240]. Through inducing mild bioenergetic stress and stimulating ketogenesis, 2-deoxyglucose treatment was found to protect neurons against degeneration in a mitochondrial toxin-based PD model [241], as well as enhance mitochondrial function and stimulate autophagic clearance of Aβ [242]. The mTOR inhibitor rapamycin-mediated enhancement of autophagy/mitophagy and AMPK activation can induce mitochondrial clearance in a number of model organisms, including *C. elegans*, *Drosophila*, and mice [243,244]. Rapamycin was also shown to reduce Aβ pathology and ameliorate cognitive dysfunction in a mutant APP transgenic mouse model [245]. Similar to rapamycin, metformin can also stimulate mitophagy through inhibitions of mTOR and complex I activities and activations of AMPK, SIRT1, and Parkin-dependent mitophagy [246,247,248]. Therefore, these observations collectively provide a strong rationale for future research into the compounds that can enhance mitophagy in AD models, such as UA, AC, and spermidine [76,249,250].

In addition, mitophagy enhancement through activation of Parkin could be another promising strategy in some disease models. Nilotinib was originally identified as a tyrosine kinase inhibitor, and was recently reported to increase Parkin abundance and ubiquitination potentially through enhancing Parkin recycling via the proteasome system [251]. Nilotinib-mediated c-ABL inhibition can also prevent tyrosine phosphorylation of Parkin, leading to the release of Parkin auto-inhibition status. Such a mechanism was demonstrated to be protective in PD models [252]. Moreover, chronic treatment with nilotinib in APP transgenic mice can enhance Aβ clearance through increasing the interaction of Parkin with Beclin 1 [253]. As for ALS and FTD, nilotinib treatment was also reported to mitigate motor and cognitive deficits in TDP-43 transgenic mice [254].

## 6. Concluding Remarks

Mitochondrial health is vital for cellular and organismal homeostasis, and mitochondrial defects have long been linked to the pathogenesis of neurodegenerative diseases such as AD, PD, ALS, HD, and others. However, it is still unclear whether cellular mechanisms required for the maintenance of mitochondrial integrity and function are deficient in these diseases, thus exacerbating mitochondrial pathology. The quality control of mitochondria involves multiple levels of strategies to protect against mitochondrial damage and maintain a healthy mitochondrial population within cells. In neurons, mitophagy serves as a major pathway of the quality control mechanisms for the removal of aged and defective mitochondria through lysosomal proteolysis. The molecular and cellular mechanisms that govern mitophagy have been extensively studied in the past decade. However, mitophagy deficit has only been recognized recently as a key player involved in aging and neurodegeneration. Given the fact that mitochondrial deficit is clearly linked to neuronal dysfunction and the exacerbation of disease defects, protection of mitochondrial function could be a practical strategy to promote neuroprotection and modify disease pathology. Mitochondrially targeted antioxidants have been proposed. In particular, the antioxidant MitoQ, a redox active ubiquinone targeted to mitochondria, has been examined and demonstrated to have positive effects in multiple models of aging and neurodegenerative disorders [255,256,257,258]. Importantly, mitophagy could be another promising target for drug discovery strategy. Therefore, further detailed studies to elucidate mitophagy mechanisms not only advance our understanding of the mitochondrial phenotypes and disease pathogenesis, but also suggest potential therapeutic strategies to combat neurodegenerative diseases.
